# Relationship Between Combined and Individual Lifestyle Components and Stress Perception in Employees: A Cross-Sectional Study Focusing on Nutrition and Physical Activity

**DOI:** 10.3390/nu18213696

**Published:** 2026-11-07

**Authors:** Gianluigi Oggionni, Giuseppina Bernardelli, Mara Malacarne, Francesco Capria, Michele Galiano, Marcello Marchese, Stefano Cribellati, Daniela Lucini

**Affiliations:** 1 BIOMETRA Department, University of Milan, 20129 Milan, Italy; gianluigi.oggionni@unimi.it (G.O.); mara.malacarne@unimi.it (M.M.); 2 Exercise Medicine Unit, Istituto Auxologico Italiano IRCCS, 20135 Milan, Italy; g.bernardelli@unimi.it; 3 DISCCO Department, University of Milan, 20122 Milan, Italy; 4 ASSIDIM, 20122 Milan, Italy; francesco.capria@assidim.it (F.C.); michele.galiano@assidim.it (M.G.);; 5 SEGE Srl, 20146 Milan, Italy; stefano.cribellati@se-ge.com

**Keywords:** lifestyle, nutrition, exercise, stress, well-being, workplace health promotion

## Abstract

**Background/Objectives**: Stress is one of the main concerns of the working population in Western countries, as it is associated with reduced well-being and performance, as well as the adoption of an unhealthy lifestyle. Nevertheless, scientific findings indicate that lifestyle improvements, particularly healthy nutrition and increased physical activity, are a pivotal tool for managing stress and improving well-being. This study aimed to verify, in a large cohort of employees, whether overall and individual lifestyle categories might be associated with different stress perceptions (primary outcome). **Methods**: In this cross-sectional study, 3380 employees from several Italian companies voluntarily completed an anonymous questionnaire on lifestyle. Five lifestyle components for which validated cut-offs are available in the literature (nutrition, physical activity, smoking, sleep, and body mass index [BMI]) were used to define lifestyle categories both individually and through a combined lifestyle score (overall lifestyle score [OLS]). **Results**: We observed that OLS healthier lifestyle categories are characterized by a progressive reduction in stress perception, which remains significant after adjustment for sex, age and job role (*p* < 0.001, ηp^2^ = 0.022). Moreover, they also showed lower fatigue and somatic symptom scores and better perceived health, sleep quality, and work performance. For individual lifestyle components, nutrition (*p* < 0.001; ηp^2^ = 0.011), physical activity (*p* < 0.001; ηp^2^ = 0.018), and sleep duration (*p* < 0.001; ηp^2^ = 0.015) were associated with stress perception, whereas findings for BMI and smoking were less consistent. **Conclusions**: Healthier dietary habits, higher physical activity volume, and a more favourable overall lifestyle profile were associated with lower self-reported stress in this population.

## 1. Introduction

Stress represents one of the main concerns of the working population in Western countries [1,2,3,4], and the total number of articles devoted to the relationship between occupational stress and employee well-being is growing at an increasing rate [3]. Scientific findings indicate that job stress is consistently associated with negative mental health outcomes such as anxiety and depression and with negative physical health outcomes, such as cardiovascular, metabolic, musculoskeletal, and gastrointestinal disorders [5,6,7,8].

Chronic stress, in fact, dramatically impairs many control mechanisms implied in the pathogenesis of many chronic diseases, such as endocrine, immunological and autonomic nervous system controls [6,7,8,9,10,11,12,13,14], and may interfere with epigenetics [15] in addition to affecting neuronal plasticity, brain resilience and mood [16].

Stress is so widespread among workers that, paradoxically, it is considered “a normal condition”; nevertheless, it dramatically impacts general well-being and job performance, resulting in a negative spiral [17]. Moreover, chronic stress is frequently associated with unhealthy lifestyle choices, such as poor nutrition, sedentariness, poor sleep, smoking, alcohol and substance abuse, exacerbating the aforementioned health problems [13,18,19]. These unhealthy behaviours may be adopted, consciously or otherwise, to cope with stress, but actually produce the opposite effect; despite providing momentary gratification, they ultimately lead to a worsening of the symptoms and an increased risk of developing or worsening chronic diseases [13,20,21]. On the other hand, many institutions propose healthy nutrition and physical activity as a convenient strategy to manage stress [1,2,4,22,23], considering that many researchers have shown that these healthy behaviours are capable of interfering positively with the different mechanisms underlying stress, such as the autonomic nervous system, immunological and endocrine controls [13,24,25,26,27,28,29,30] and even epigenetics [15].

This bidirectional relationship between stress, nutrition, and physical activity is intriguing and should be considered when designing workplace intervention programs to foster well-being [21].

Psychosocial risks and the resulting negative consequences are among the most challenging issues in occupational safety and health. Besides their detrimental effect on individual health, they can also negatively impact the efficiency of organizations, as well as national economies [4]. They may arise due to poor workplace organization and/or poor social context in the workplace (such as excessive workloads, conflicting demands and lack of role clarity, job insecurity, lack of support from management or colleagues, and a lack of influence over the way the job is done [1]) as well as personal issues. From a “medical point of view,” while it is quite difficult to act on organizational and managerial work-related aspects, it is possible to act on the individuals to counteract the physiological and psychological effects of stress, increasing the personal resources needed to manage stressors [13].

Emerging data suggest that healthy behaviours may help in this regard. They may counteract negative stress-related effects. Nutrition, for instance, helps to modulate stress perception and mental health by acting on the gut–brain axis and influencing the microbiota [24,25]. Healthy diets, such as the Mediterranean diet, may also act as anti-inflammatory agents, reducing low-grade inflammation, improving the composition of gut microbiota and improving neurotransmitter production [24,25,31]. In addition, healthy nutrition (particularly when paired with tailored exercise programs) plays an indispensable role in body weight control, helping to reduce the negative consequences linked to overweight and obesity [13]. Physical activity, particularly aerobic exercise, has been shown to reduce stress, directly improving autonomic nervous system control [32], reducing inflammation [28], and improving metabolic control [33]. Moreover, clinical and pre-clinical neuroscience research [29,30] has explored the neuroprotective mechanisms of endurance exercise and identified cardiorespiratory fitness as a crucial factor in promoting healthy brain ageing, supporting brain resilience and function, and improving cognition. Aerobic exercise, in fact, may improve cerebral blood flow, reduce inflammation, and enhance neuroplasticity [29,30].

Of particular significance is the observation that various healthy lifestyle behaviours, including proper nutrition and regular exercise, do not operate in isolation; rather, the adoption of multiple healthy lifestyle behaviours may produce effects that exceed the sum of their individual effects [34,35,36]. The World Health Organization recently proposed guidelines [35] on the employment of the association of physical activity and the management of waist circumference in cancer patients, underlying their positive cumulative effect; a recent scoping review on the combined impact of nutrition and physical activity on students’ well-being supports the use of multicomponent strategies over single-behaviour initiatives [36].

In previous papers [37,38], we observed that employees who meet current WHO physical activity recommendations are characterized by a lower perception of stress and that age and sex may influence stress perception and lifestyle. In this paper, we hypothesized that different categories of lifestyle may be associated with different levels of stress perception, focusing more on overall lifestyle than on a few of its components. The goal of the present research is to verify, in a large cohort of Italian employees, whether progressively healthier lifestyle categories (obtained both independently and as a combined lifestyle score) might be associated with different perceptions of stress, which represents our primary outcome. Moreover, associations with other health-related variables were examined as secondary outcomes. These data might help to clarify the relationship between stress and mental and physical health, supporting the development of tailored workplace health-promotion strategies.

## 2. Materials and Methods

In this cross-sectional study, a group of 3380 employees (aged 20–88 years) of several Italian companies voluntarily filled out an anonymous lifestyle-related questionnaire which was available from January 2021 to May 2024 on the web page of ASSIDIM (a non-profit association that provides the associated companies and their employees’ families with financial assistance and support in case of accident, disease, death, and invalidity), which, since its foundation in 1981, has been on a mission to promote health among workers and associated companies.

The questionnaire was anonymous and was posted on the ASSIDIM website (www.assidim.it, accessed on 6 November 2026), which was open to all employees (and their family members) of the companies associated with ASSIDIM; it was not possible, for privacy reasons, to know the name, the location, or the number of companies whose employees participated or how many employees were potentially eligible.

We recently described [37,38,39] the methodology used to develop this questionnaire, which was validated through statistical analysis [40]. It provided every single participant with a personalized, immediate report based on the information entered to improve the reliability of the inserted information. The questionnaire collected data on lifestyle (diet habits, physical activity, smoking, sleep hours, and perceptions of stress, fatigue, and stress-related somatic symptoms), job category, perceived quality of personal health, sleep, and job performance, and anthropometric data.

All subjects who participated voluntarily in the survey provided their information anonymously via the questionnaire. They were aware that the collected data could possibly be used in an anonymized and aggregated form for scientific purposes. This research was part of an ongoing study on the role of questionnaires in assessing lifestyle approved by the local Ethical Committee (Istituto Auxologico Ethical Committee, code 2023_04_18_14 dated 4 May 2023).

### 2.1. Lifestyle Assessment and Other Data Considered in the Questionnaire

As we stated in our previous papers (see references [37,38,39,40] for details), we assessed lifestyle considering the following items:

- Physical activity (total activity volume) was assessed by the short version of the International Physical Activity Questionnaire (IPAQ) [41], a validated questionnaire that is frequently employed in scientific research to assess the volume of performed physical activity, which focuses on intensity (nominally estimated in Metabolic Equivalents—METs—according to the type of activity) and duration (in minutes) of physical activity. We considered for the analysis the following levels: activities of moderate intensity (≈4.0 METs) and activities of vigorous intensity (≈8.0 METs). These levels were used to calculate the weekly physical activity volume of self-reported moderate–vigorous physical activity (METsMV; MV—moderate and vigorous) using the following equations:
  METsMV = 4 × M × dM + 8 × V × dV,(1)
  where METsMV stands for weekly moderate and vigorous physical activity volume expressed in MET·minutes/week; M is the number of minutes/days of moderate-intensity activity; dM is the number of days/weeks of moderate-intensity activities; V is the number of minutes/days of vigorous-intensity activity; and dV is the number of days/weeks of vigorous-intensity activities. The quantity in (1) may then be considered the total weekly volume of moderate and vigorous physical activity. We did not consider in the total volume of physical activity the time dedicated to walking activities to avoid possible misinterpretation of this parameter, considering the self-reported nature of the questionnaire.
- Nutrition was assessed using the American Heart Association (AHA) Diet Score [42,43,44], taking into consideration the following elements: Fruit/vegetables, fish, sweetened beverages, wholegrain and sodium consumption (the assessment of the latter was adapted to Italian eating habits [37,38,40]). The final score reflects the number of healthy elements the subject reached [44] and is considered a proxy for “nutrition quality”.
- Perception of stress, fatigue and subjective somatic stress-related symptoms (4SQ) was assessed using a self-administered questionnaire [40] providing nominal self-rated Likert scales from 0 (“no perception”) to 10 (“highest perception”) for each measure. We used a short version of the 4SQ that included four somatic symptoms (perception of heart beating, perception of breathing, perception of muscle tension, and perception of a knot in the stomach); thus, the total score ranged from 0 to 40.
- Smoking: we classified as non-smokers all those who reported never having smoked or having quit more than a year ago [45].
- Sleep: asking for the number of hours slept [42].
- Usage of alcohol: considering Italian habits, asking the number of glasses of wine (125 mL) or beer (330 mL) consumed per week and the number of glasses of spirits (40 mL) consumed per week, considered as 1 alcoholic unit, corresponding approximately to 12 gr of alcohol [46].
- Perception of sleep and health quality was assessed by providing ordinal self-rated 11-point Likert scales from 0 (“very bad”) to 10 (“very good”) for each measure. Similarly, job performance perception was assessed using ordinal self-rated 11-point Likert scales from 0 (“very bad”) to 10 (“very good”) [38,39].
- Job role: Executives, middle managers, non-managerial employees and retired workers were considered.

### 2.2. Overall Lifestyle Score

To account for the comprehensive cumulative effect of all the lifestyle components together, we considered a combined lifestyle score (overall lifestyle score—OLS). It was built considering the lifestyle behaviours or factors listed in the international literature [42,43,45], which were possible to collect using a web-based questionnaire and for which it was possible to have validated cut-offs [42,43,45] corresponding to different levels of health status. These lifestyle components are as follows: nutrition, physical activity, smoking status, hours of sleep and body mass index (calculated from reported weight and height). Initially, these components were used to calculate categories corresponding to poor, intermediate or ideal health, as suggested by validated international literature [42,43,45,47] (see Table A1). For each component, we assigned points according to the corresponding category (whereas higher points indicated healthier behaviour): poor = 0 points; intermediate = 1 point; ideal = 2 points. The OLS was then calculated by summing the points assigned to the five lifestyle components, resulting in a score ranging from 0 to 10, with higher scores indicating a healthier overall lifestyle profile. Based on the observed distribution of OLS values in the study population, participants were classified into three ordered categories: POOR (OLS 0–5), INTERMEDIATE (OLS 6–7), and IDEAL (OLS 8–10), representing progressively healthier overall lifestyle profiles.

### 2.3. Statistics

**Figure 1:**
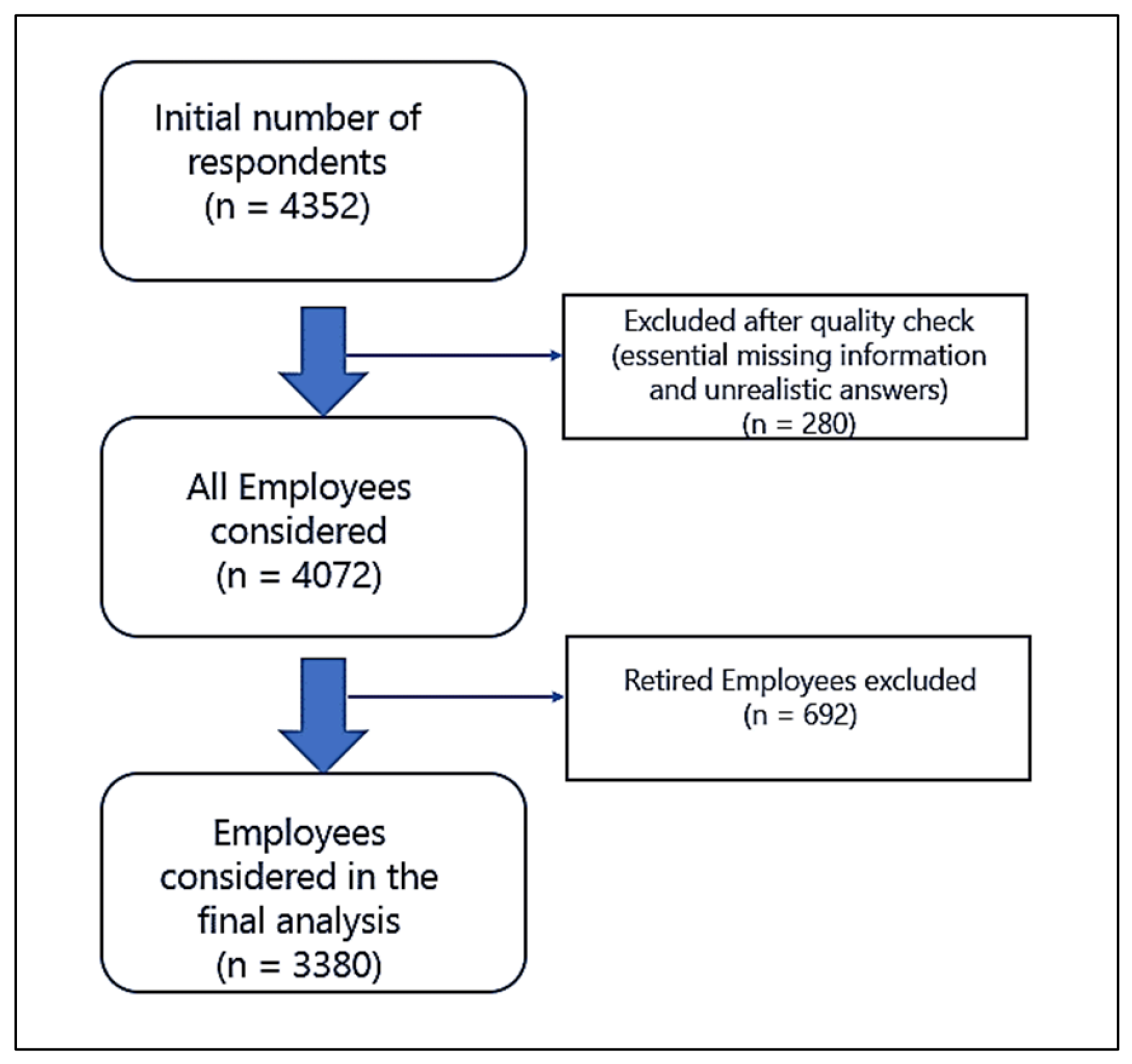
Flowchart of study participants.

A quality check of collected data was performed to remove subjects with essential missing information and unrealistic answers from the original dataset (n = 4352). The initial dataset of validated data consisted of records of 4072 employees; from this dataset, we removed subjects who declared themselves to be retired from an active role, and a total of 3380 were considered in the final analysis of the present investigation (see Figure 1). Our goal was to consider in the analysis subjects who filled out the entire questionnaire with realistic answers and were working in an active job role.

A sensitivity power analysis was conducted to estimate the minimum detectable effect size for the main effect of OLS categories in the ANCOVA model, with stress perception as the primary outcome.

With 3380 participants, the study had 80% power at α = 0.05 to detect effects as small as Cohen’s f = 0.053 (ηp^2^ = 0.0028) [48].

Continuous variables are presented as the mean ± standard deviation (SD). Categorical variables are reported as absolute frequencies and percentages. Differences between females and males were assessed using independent-samples t-tests for continuous variables and Pearson’s chi-square test for categorical variables. Differences across occupational roles in continuous variables were assessed using pairwise comparisons of column means based on two-sided t-tests assuming equal variances, with Bonferroni correction for multiple comparisons. Categorical variables were compared using Pearson’s chi-square test.

The primary analysis examined differences in stress perception across overall lifestyle score (OLS) categories using analysis of covariance (ANCOVA). The same model structure was applied to the six secondary outcomes: fatigue, 4SQ, perceived health, sleep quality, work performance, and alcohol consumption. The three-factor ANCOVA analyses incorporated OLS category, sex, and job role as fixed factors, with age serving as a covariate. All two-way interactions, along with the three-way interaction among OLS category, sex, and job role, were included in each model to determine whether the relationships between OLS categories and study outcomes varied by sex and job role. Separate, three-way ANCOVA models were conducted for each of the five individual lifestyle components included within the OLS (namely, the AHA Healthy Diet Score, physical activity, smoking status, sleep duration, and BMI). In these models, OLS category was replaced by the corresponding lifestyle component category, with each one classified as poor, intermediate, or ideal for each lifestyle component based on the criteria detailed in Table A1. These models included the lifestyle component category, sex, and job role as fixed factors, with age as a covariate; we also included all two-way interactions and the three-way interaction between lifestyle component category, sex, and job role.

An additional model for stress perception included all five lifestyle components simultaneously, together with age, sex, and occupational role, to assess whether the associations with nutrition and physical activity persisted after accounting for the other components.

Pairwise comparisons between lifestyle categories were based on model-adjusted estimated marginal means, with Bonferroni adjustment of *p*-values and 95% confidence intervals. Effect sizes for the main category in ANCOVA models were evaluated using partial eta squared (ηp^2^) and reported alongside *p*-values. Given the large sample size, statistical significance was interpreted together with the magnitude of the observed effects [48]. To account for multiple comparisons ANCOVA *p*-values were adjusted using the false discovery rate (FDR) according to the Benjamini–Hochberg procedure [49].

ANCOVA diagnostics included examination of standardized residual distributions, Q–Q plots, residual-versus-age and residual-versus-predicted plots, and Cook’s distance to assess residual normality, potential nonlinear patterns with age, and influential observations. Stress perception and fatigue were measured using 0–10 numerical rating scales and treated as approximately continuous outcomes to estimate adjusted mean differences. This approach was considered reasonable given the 11 response levels and large sample size, while acknowledging that these measures are ordinal and bounded. To evaluate robustness to potential heteroskedasticity, sensitivity analyses using HC3 robust standard errors were conducted for all seven outcomes, and results were compared with those from conventional ANCOVA analyses.

Given the ordinal nature of the poor, intermediate, and ideal categories, trends across these categories were assessed using the Jonckheere–Terpstra test, whereas differences among categories were evaluated through the Kruskal–Wallis test. These complementary analyses did not adjust for age, sex, or occupational role.

Finally, as a sensitivity analysis, the analyses were repeated with OLS treated as a continuous score (0–10), while sex and job role were retained as fixed factors and age as a covariate. All statistical tests were two-sided, and a *p*-value less than 0.05 was deemed statistically significant. All analyses were performed using IBM SPSS Statistics, version 30.0 (IBM Corp., Armonk, NY, USA). The sensitivity power analysis was conducted in R (version 4.6.1; R Foundation for Statistical Computing, Vienna, Austria).

## 3. Results

### 3.1. Descriptive Results

Table 1 reports the characteristics of the overall study population (n = 3380) subdivided into females (n = 1556) and males (n = 1824). Significant sex-related differences were observed; in particular, female employees were younger; they reported a higher perception of stress, fatigue and stress-related somatic symptoms (4SQ score), and a reduced quality of health and sleep. Females were also characterized by higher-quality nutrition and a reduced volume of physical activity. Considering occupational roles, executives (employees in senior leadership roles) accounted for 38.0% of male employees and 15.9% of female employees. Middle managers (workers who carry out jobs with a relative degree of responsibility) accounted for 36.9% of male employees and 31.2% of female employees. Non-managerial employees (1244 office workers and 37 blue-collar workers, performing operational roles with less responsibility) accounted for 25.1% of male employees and 53.0% of female employees.

Table A2 reports characteristics of the study population, subdivided by job role. Non-managerial employees (office or blue-collar workers) were characterized by higher perceptions of stress, fatigue, and stress-related somatic symptoms; lower perceptions of work performance, a lower AHA Diet score and lower physical activity.

### 3.2. Adjusted Analyses, Interaction Effects, and Sensitivity Analyses

Data regarding sex, age and job role differences in stress perception have already been presented in the scientific literature [38,50,51] and need to be accounted for while examining the relationship between lifestyle and stress perception. Consequently, we performed the next statistical analysis (ANCOVA models), including age as a covariate and sex and job role as fixed factors.

Table 2 shows the association of overall lifestyle score categories with health variables.

**Figure 2:**
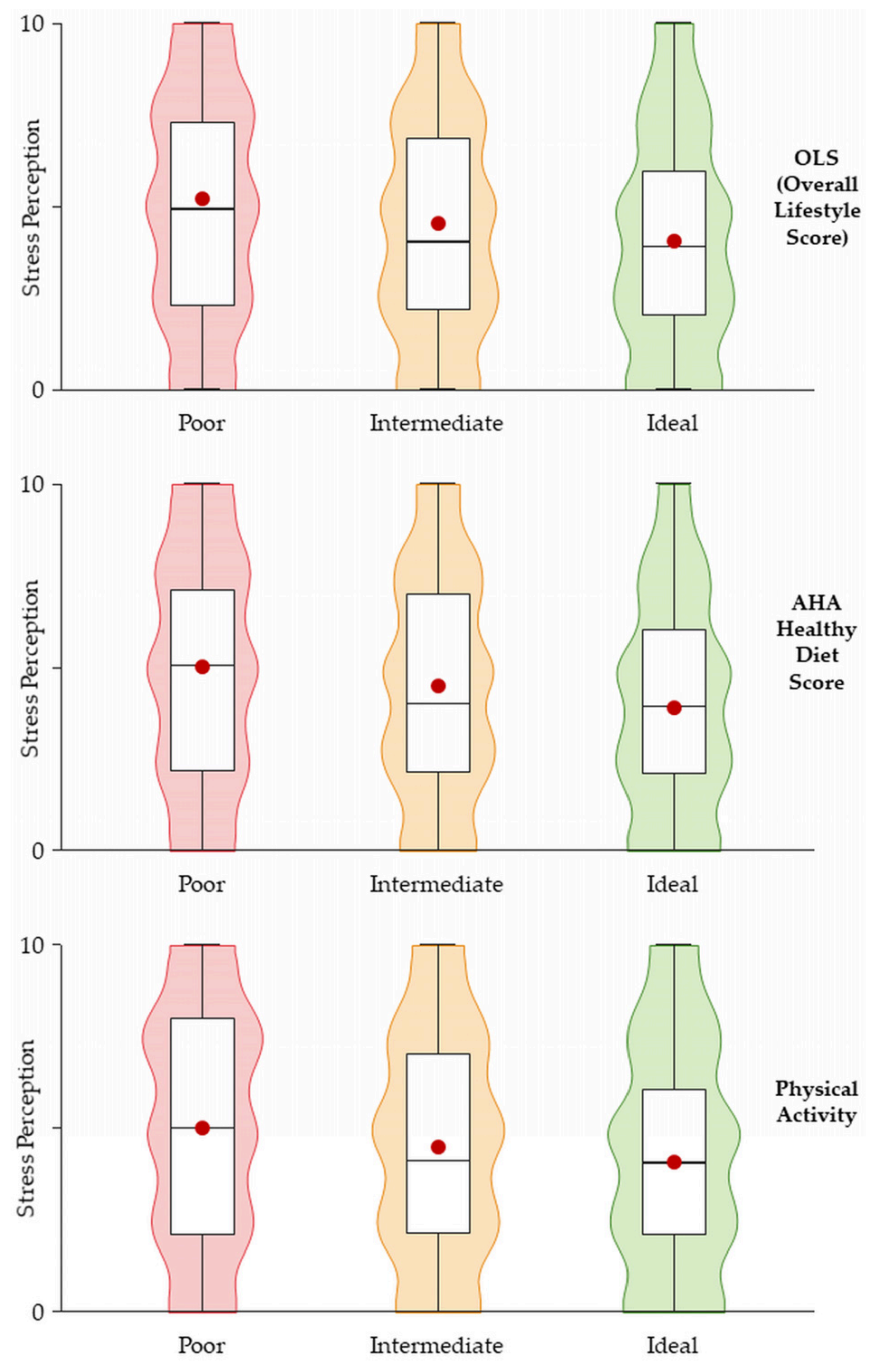
Violin plots showing the distribution of stress perception across poor, intermediate, and ideal categories of the overall lifestyle score (OLS; (**top panel**)), AHA Healthy Diet Score (**middle panel**), and physical activity (**bottom panel**), respectively. Violin widths represent the distribution of individual observations; white boxes indicate the interquartile range; horizontal black lines the median; and red dots the mean. Stress differed significantly across the OLS, AHA Healthy Diet Score and physical activity categories (*p* < 0.001). All pairwise comparisons (poor vs. intermediate, poor vs. ideal, and intermediate vs. ideal) were significant for OLS, AHA Healthy Diet Score and physical activity (*p* < 0.05). Statistical comparisons were performed using ANCOVA models including lifestyle category, sex, and occupational role as fixed factors and age as a covariate. All pairwise comparisons between categories were statistically significant based on model-adjusted estimated marginal means with Bonferroni correction.

Employees with a healthier lifestyle report lower stress, fatigue, and stress-related somatic symptoms, as well as better perceptions of work performance and health, and these effects remain significant after adjusting for sex, age, and job role (see Figure 2).

Diagnostic plots did not reveal pronounced nonlinear patterns with age. Deviations from residual normality were generally modest and mainly involved the distribution tails, although departures were more pronounced for some secondary outcomes (perceived health and alcohol consumption). Cook’s distance values were low across variables (mean 0.0003; maximum 0.04). Sensitivity analyses using HC3 robust standard errors yielded findings consistent with the conventional analyses, with no substantial changes in the principal conclusions.

Additional (sensitivity) analysis treating OLS as a continuous variable (score 0–10) confirmed the main findings: OLS remained significantly associated with stress (*p* < 0.001, ηp^2^ = 0.037), fatigue (*p* < 0.001, ηp^2^ = 0.047) and 4SQ (*p* < 0.001, ηp^2^ = 0.029).

Table 3 and Table 4 report how stress perception and other variables vary across categories (see Table A1), based on the five components used to calculate OLS (AHA Healthy Diet score, physical activity, smoking habits, sleep, and BMI). Briefly, subjects belonging to healthier nutrition categories (category: ideal; score 4–5) (Table 3a) are characterized by a progressive reduction in stress perception, in addition to reduced perception of fatigue and somatic symptoms, improved perception of quality of health and of sleep, and reduced use of alcohol. Similar results were observed when considering physical activity categories (Table 3b)—more active subjects reported lower perception of stress and other considered variables, which changed significantly across the three physical activity categories. We conducted an additional ANCOVA that included all five lifestyle components in the same model and adjusted for age, sex, and occupational role. After adjusting for the other lifestyle components, AHA Diet score and physical activity remained significantly associated with stress perception (AHA Diet score: *p* < 0.001, ηp^2^ = 0.009; physical activity: *p* < 0.001, ηp^2^ = 0.011). Although the effect sizes were slightly smaller than in the separate analyses, both associations remained statistically significant.

Associations with stress perception varied across sleep, smoking, and BMI categories (Table 4). Sleep duration showed a more consistent pattern, while BMI showed a non-monotonic association, and smoking status was not significantly associated with stress perception in the adjusted ANCOVA models.

Interestingly, we observed that the three BMI categories present no significant differences regarding the mean value of the AHA Healthy Diet score, respectively, of 2.25 ± 0.97 in subjects characterized by BMI ≥ 30 kg/m^2^, 2.18 ± 1.01 in subjects with a BMI ≥ 25–29.9 kg/m^2^, and 2.29 ± 1.01 in subjects with a BMI less than 25 kg/m^2^ On the contrary, significant differences (*p* < 0.001) were observed for physical activity volumes. In particular, obese subjects were less active than the other groups (*p* < 0.001, respectively, of 402.70 ± 920.05 MET·min/week in subjects characterized by BMI ≥ 30 kg/m^2^; 714.17 ± 979.58 MET·min/week in subjects characterised by a BMI ≥ 25–29.9 kg/m^2^; and 806.23 ± 1051.54 MET·min/week in subjects characterized by a BMI less than 25 kg/m^2^.

### 3.3. Trend Analysis

Table 5 reports the trend test. Notably, a significant trend is present for almost all considered variables (*p* < 0.001). In particular, stress perception progressively decreases across both combined (OLS) and individual nutrition, physical activity, and sleep lifestyle components.

## 4. Discussion

In this study of a large population of Italian employees, we observed that healthier lifestyle categories (based on a combined overall lifestyle score [OLS]) are associated with a progressive reduction in stress perception, fatigue, and other stress-related somatic symptoms, which remain significant after adjusting for sex, age, and job role. In addition, these categories are distinguished by improved perceptions of sleep, health quality, and work performance. When considering individual lifestyle components, stress perception decreased progressively across healthier nutrition, physical activity, and sleep categories, whereas BMI and smoking showed less robust patterns. These results were consistent after FDR correction.

These findings may garner better support for health-related policies and practices in workplaces, corroborating the possibility of using healthy lifestyle choices, particularly healthy nutrition and physical activity, as strategies to improve well-being. However, given the cross-sectional nature of this investigation, we may not establish the direction of the relationship between lifestyle and stress perception.

Stress makes up the second most common work-related health problem affecting European workers [4], and the percentage of employees who report facing risk factors that can adversely affect their mental health is nearly 45% [4], while in 2026, 40% of employees worldwide said they had experienced “a lot of stress” [2]. On the other hand, stress also represents a real issue in the medical field, being considered an independent risk factor for many chronic diseases such as cardiometabolic diseases and cancer [5,6,7,8,9,10,11,12], and negatively affecting most of the mechanisms responsible for those conditions, such as immunological, hormonal and autonomic nervous system controls and even epigenetic mechanisms. Moreover, stress may also indirectly increase cardiometabolic/oncological risk, leading people to favour unhealthy lifestyle choices such as poor nutrition, sedentariness, smoking and substance abuse [13,18,19]. Notably, the negative effects of stress relate not only to the increased risk of mental or chronic diseases that may appear in the future, but also to reduced well-being and academic or work performance [4,13,52,53] in the present, with clear health and economic consequences [1,2,3,4,52]. The workplace may be an ideal context for applying stress-management policies, offering advantages for both the individual and the company, including improved performance, productivity, safety, reduced absenteeism, and an enhanced corporate climate [54,55]. Strategies may include improving working conditions, organization, management of relationships, and taking action to improve the workplace climate and communications [4]. In fact, we have to consider that workload is only one kind of workplace stressor, and sometimes other stressors (such as role ambiguity, lack of information about job execution or how one’s work will be evaluated, role conflict, and difficulties in determining priorities) may be determinant, particularly in non-managerial employees [1]. In addition, workplace stress is influenced by organizational, psychosocial, and individual factors. Unfortunately, it is not possible to tackle all work stressors, especially since many relate to people’s personal lives outside work and to an individual’s ability to cope [6,13,14,20]. Hence, improving individuals’ access to resources that will help them cope with stress may be another important strategy to consider. Among these strategies, lifestyle improvement has been shown to play a significant role [22,23]. Nevertheless, many factors may influence the relationship between stress and lifestyle, particularly age, sex, and job role [1,2,50,56].

For instance, female subjects report higher stress perception and fatigue; non-managerial employees (frequently more represented in the female population) report higher stress levels [1,57], and age may significantly influence coping strategies [38,56]. In a previous paper, we observed that young employees report higher stress perception and a worse lifestyle [38]. In the present research, we extended this finding, showing that the combined OLS was associated with progressively lower stress perception, fatigue, and stress-related somatic symptoms. When analyzed separately, however, lifestyle components exhibited heterogeneous patterns of association after adjustment for sex, age, and job role. Data in the recent literature [36] on the combined contribution of nutrition and physical activity to psychological well-being corroborate the observed relationship between OLS and stress.

Considering the confounding effects of age, sex and job role could help to improve our understanding of some inconsistencies present in the literature regarding the relationship between lifestyle and stress [55,58].

Obviously, we have to consider the double link between stress and lifestyle [21]. On one side, stress may induce the adoption of unhealthy lifestyles; on the other, healthy lifestyles foster well-being and reduced stress perception. In the present research, we may not have disentangled this issue. However, the literature offers many examples of interventions based on lifestyle improvements capable of reducing stress perception [1,2,4,21,22,23,24,25,26,27,32,52,55,59] and the negative effects of stress on the mechanisms responsible for disease, health, longevity and well-being, suggesting that stress and many chronic diseases and poor well-being share etiopathogenetic mechanisms that could potentially be improved by salutogenic behaviours [5,6,7,8,9,10,11,12,32].

The possibility of having a clear picture of employees’ lifestyles is an essential step in defining the needs and characteristics of a working population and, therefore, designing a tailored intervention program to reduce stress and foster health and well-being at the worksite. In this research, we used an anonymous web-based questionnaire designed to collect information on lifestyles. In a previous paper using this tool, we observed interesting data on the relationship between stress and physical activity [37], the role of age in determining lifestyles [38], and the specific need to improve the studied population’s lifestyle [39]. In addition, using a sophisticated statistical approach based on classification trees [39], we unveiled specific subject typologies characterized by a greater likelihood of a specific desire or behaviour. Interestingly, young female employees were the group most likely to express a desire to improve their stress management, and exercise was chosen as a strategy to manage stress independently of age and sex. Moreover, specific stress-management strategies (such as psychological intervention) were preferred only by already fit subjects who reported a high level of stress perception [39]. The present investigation adds new information to that reported in our previously published studies, and its novelty is the specific focus on overall lifestyle, instead of focusing on a few of its components, and the observation of a different perception of stress, taking into account three different lifestyle categories (both considering combined and individual lifestyle components), which remained consistent after adjustment for sex, age and job role. We also have to specify that the study population of the present study is quite different from the populations considered in the previous investigations, as it is related only to active workers, excluding retired ones. The first published paper showed [37] the relationship between physical activity and stress; the second paper [38] reported the influence of age and sex on lifestyle and stress perception; the third [39] analyzed specific subject typologies and their desire and attitude to change behaviour.

The questionnaire employed in the present paper was also used in different populations, such as university students [60], university employees [61] and patients [62,63], and it could define specific behavioural characteristics that may help to tailor intervention programs to foster well-being and health. Moreover, we observed significant correlations between questionnaire-derived parameters (such as physical activity volume, stress perception, and nutrition quality) and physiological parameters such as markers of autonomic control [32,62,63,64,65]. In particular, stress perception showed a significant inverse correlation with cardiopulmonary fitness [66], and a direct association with the marker of sympathetic activity [32]. In the present study, stress perception decreased progressively across combined OLS categories after adjustment for sex, age, and job role. Associations with individual components were more heterogeneous, highlighting the importance of distinguishing the overall lifestyle profile from its separate components. The OLS and selected components were also associated with other outcomes, including perceived work performance, health, and sleep quality.

The possibility of unveiling such information with regard to a specific working population might be useful as a motivational tool to help workers adopt healthier behaviours, underlining the benefits of stress reduction, well-being and work performance improvements—benefits which may be obtained in the present. Frequently, the benefits of healthy nutrition, physical activity, not smoking, etc., are linked to the prevention of chronic diseases; this benefit, albeit important from a preventive point of view, may be less appealing for some categories of employees, such as young people or people who experience reduced well-being due to many stressors that are present in their lives. Notably, these subjects have less healthy lifestyles [67,68] and participate less in prevention campaigns limited to medical examinations (check-ups) [69,70], which fail to prevent chronic diseases without lifestyle and risk changes.

Healthy nutrition deserves a comment; in this paper, we observed a relationship between nutrition quality and stress perception. We used a simple tool, the AHA Healthy Diet score [42,44], which assesses nutrition quality through five questions about consumption of fruit and vegetables, fish, sugar-sweetened beverages, wholegrain foods, and salt. We have to recognize the impossibility of using this score to analyze aspects that link stress and nutrition, like hypoglycaemia provoked by nutritional mistakes. Moreover, this score presents some important limitations, considering a limited number of dietary indicators rather than a comprehensive nutritional evaluation. Nevertheless, a more detailed nutrition assessment was outside the scope of the present study. Interestingly, employees characterized by a BMI less than 25 kg/m^2^ did not present an ideal value of the AHA Healthy Diet score (score 4–5), but a score of 2.29 ± 1.01, corresponding to “intermediate health [42,43,44]. A similar pattern has also been observed in other populations, including university students [60] and university employees [61]. The finding that this group has suboptimal nutrition quality opens the door to a different approach to preventive strategies, as these are usually based on fighting overweight and obesity by promoting healthy nutrition to manage these conditions and imply that if one’s weight is normal, there is no need to improve one’s diet. In contrast, intervention programs might more specifically address the importance of nutrition quality (independently of body weight) and educate people to adhere to healthy nutrition patterns that support maintaining a normal body composition. Another interesting point is that the association between BMI category and stress perception does not follow a simple progressive pattern in which lower BMI consistently corresponds to lower stress perception. We have to comment that this finding should be interpreted cautiously, particularly considering the unequal distribution of participants across BMI categories and the relatively small number of individuals with obesity (n = 215), which may affect the precision of the estimates. Moreover, in the group of participants characterized by a BMI below 25 kg/m^2^, there might be employees presenting with a very low BMI—a condition which may be associated with increased stress perception. Moreover, the significant interaction between BMI category and sex for stress perception suggests potential sex-related differences in this association, which deserve further investigation in specifically designed studies.

Finally, we have to comment on the discrepancy between the significant unadjusted trend for smoking status and stress perception (*p* = 0.005) and the non-significant adjusted ANCOVA (*p* = 0.702). This may reflect differences in covariate adjustment and statistical methodology. While the Jonckheere–Terpstra test evaluates ordered trends across categories without covariate adjustment, ANCOVA assesses category differences while accounting for age, sex, and occupational role. Therefore, greater emphasis was placed on the adjusted results when interpreting associations between smoking status and stress perception.

*Limitation*: This study presents some limitations.

First, the cross-sectional design of this study does not permit us to draw conclusions about causality or long-term effects, and longitudinal and interventional studies are necessary to understand the long-term impact of lifestyle on stress perception.

Second, data obtained through self-reported questionnaires might be of suboptimal quality. To address this, we provided participants with a personalized, immediate report based on the filled information, increasing their compliance [40] to provide trustworthy data on their present condition. Moreover, because the questionnaire was completed voluntarily, sampling selection bias is expected, limiting the generalizability of the results to the overall employee population. Nevertheless, the number of respondents was relatively high for this investigation, and we observed a wide range of scores across all questions. We also recognize that assessing stress perception using a single self-reported numerical scale rather than more widely established multidimensional instruments may have influenced the observed associations and limited the quality and generalizability of the findings.

Third, we did not account for all potential confounding variables that could affect stress perception or the coping strategies participants ultimately used. We assessed only stress, fatigue, and stress-related somatic symptoms using very simple scales, considering the need to assess stress perception in a simple and cost-convenient manner. Nevertheless, we have to consider that excessively long questionnaires can compromise the validity and reliability of the collected data [71]. The feasibility of assessing stress perception simply using a few questions inquiring about stress from both a somatic (asking questions regarding the perception of fatigue and other somatic symptoms such as palpitations or muscular tension) and a cognitive (directly asking about stress perception: “do you feel stressed” [72]) point of view may provide a simple metric for workplace health-promotion interventions [32,38,39,40].

Fourth, we did not explore the underlying mechanisms driving the relationship between stress perception and lifestyle, such as neurocognitive, psychological and physiological factors.

Fifth, some components were unevenly distributed across categories, particularly BMI, smoking, and sleep. Specifically, the least represented groups included participants with poor BMI (n = 215), intermediate smoking status (n = 60), and poor sleep (n = 118). Although the large overall sample size provided substantial statistical power, the smaller subgroup sizes may have reduced the precision of component-specific estimates and should be considered when interpreting these analyses.

Sixth, although the thresholds used to categorize the OLS were based on the observed score distribution and have not been externally validated, sensitivity analysis treating the OLS as a continuous score yielded consistent findings for stress perception, reducing the likelihood that the observed association was driven by the selected category thresholds.

We want to emphasize that these thresholds were based on the observed distribution of scores in the study population and should not be interpreted as externally validated clinical thresholds. Moreover, we have to clarify that our analyses on OLS do not directly demonstrate biological or statistical synergy among lifestyle components and the effect sizes observed for the main associations remain relatively small.

Seventh, we assessed physical activity and other lifestyle components using only a few questions in a web-based questionnaire, which may have limited response reliability and is consistent with the possibility of recall bias due to the questionnaire’s self-reported nature; more objective measures or a more detailed questionnaire might have improved data quality.

Eighth, the recruitment strategy used in the present investigation may affect the representativeness and generalizability of the findings. Moreover, given the number of outcomes and statistical comparisons examined, consider the potential for type I error when interpreting the findings. We applied a Bonferroni correction to post hoc analyses to account for multiple comparisons between lifestyle categories. Nevertheless, results should be interpreted not based on statistical significance alone, but also in light of the magnitude of the observed associations and the consistency of findings across health- and work-related outcomes.

## 5. Conclusions

Healthier dietary habits, a higher volume of physical activity, and a more favourable overall lifestyle profile were associated with lower self-reported stress in this population.

OLS healthier lifestyle categories are characterized by a progressive reduction in stress perception (*p* < 0.001), which remains significant after adjustment for sex, age and job role. Moreover, they were also associated with less perceived fatigue and somatic symptoms, improved quality of health and job performance perceptions. Associations varied across individual lifestyle components. Healthier nutrition, greater physical activity, and more favourable sleep duration were associated with lower stress perception, whereas BMI showed a non-monotonic pattern and smoking status was not significantly associated with stress in adjusted analyses.

Understanding these relationships might be useful for tailoring interventions based on improvements in nutrition and physical activity to foster well-being and health in the workplace. We have to recognize, nevertheless, that our findings could benefit from further analysis with a more detailed methodology, which might be considered when designing future studies. For instance, the introduction of a more extensive assessment of stress perception, sources of job stress, coping strategies, and social and economic aspects may improve our knowledge about the relationship between stress and lifestyle.

## Figures and Tables

**Table 1:** Characteristics of the study population overall and by sex.

Variable	All (N = 3380)	Females (n = 1556)	Males (n = 1824)	*p* Value
	Mean ± SD	Mean ± SD	Mean ± SD	
**Overall lifestyle- and health-related variables**				
Stress P.	4.48 ± 2.97	5.22 ± 2.99	3.85 ± 2.81	<0.001
Fatigue P.	4.29 ± 2.98	5.15 ± 3.05	3.56 ± 2.72	<0.001
4SQ Score	7.00 ± 7.49	9.07 ± 8.18	5.23 ± 6.34	<0.001
Alcoholic units/week	1.63 ± 1.94	1.16 ± 1.66	2.03 ± 2.07	<0.001
Work performance	6.56 ± 2.22	6.50 ± 2.16	6.62 ± 2.27	0.117
Perceived health	7.03 ± 1.50	6.95 ± 1.51	7.10 ± 1.49	0.004
Sleep quality	6.35 ± 2.00	6.25 ± 2.06	6.44 ± 1.95	0.005
**Lifestyle components**				
Sleep duration [hours/night]	6.64 ± 1.08	6.67 ± 1.15	6.62 ± 1.02	0.182
AHA Healthy Diet Score [a.u.]	2.26 ± 1.01	2.32 ± 1.02	2.20 ± 1.00	0.001
Physical Activity (METs MV) [MET·min/week]	754.49 ± 1028.24	610.65 ± 912.34	877.21 ± 1103.16	<0.001
Smoking status [n (%)]				0.343
- Current smoker	427 (12.6)	209 (13.4)	218 (12.0)	
- Former smoker ≤12 months	60 (1.8)	30 (1.9)	30 (1.6)	
- Non-smoker	2893 (85.6)	1317 (84.6)	1576 (86.4)	
**Sociodemographic and anthropometric characteristics**				
Age [years]	49.25 ± 10.91	46.72 ± 10.20	51.42 ± 11.03	<0.001
Weight [kg]	71.83 ± 14.54	61.97 ± 10.96	80.25 ± 11.66	<0.001
Height [cm]	172.19 ± 9.09	165.39 ± 6.36	178.00 ± 6.74	<0.001
Waist circumference [cm]	86.66 ± 14.64	79.32 ± 13.21	92.93 ± 12.75	<0.001
BMI [kg/m^2^]	24.08 ± 3.71	22.65 ± 3.76	25.30 ± 3.19	<0.001
**Occupational role [n (%)]**				<0.001
Executives	941 (27.8)	247 (15.9)	694 (38.0)	
Middle managers	1158 (34.3)	485 (31.2)	673 (36.9)	
Non-managerial employees	1281 (37.9)	824 (53.0)	457 (25.1)	

**Abbreviations:** n = number of participants in each subgroup; SD = standard deviation; P. = perception; 4SQ Score = somatic stress-related symptom score; AHA = American Heart Association; a.u. = arbitrary units; MET = metabolic equivalent; MV = moderate-to-vigorous physical activity; min = minutes; h = hours; BMI = body mass index; kg = kilograms; cm = centimetres; m^2^ = square metres; work performance = self-rated work performance. Continuous variables are presented as mean ± SD; smoking status and occupational role are presented as n (%). *p*-values for continuous variables are from two-sided comparisons between females and males; smoking status and occupational role were compared by Pearson’s chi-square test.

**Table 2:** Associations of overall lifestyle score categories with health variables.

Overall Lifestyle Score (OLS)	Poor Mean ± SD n = 669	Intermediate Mean ± SD n = 1429	Ideal Mean ± SD n = 1282	*p* (Cat.)	ηp^2^	*p* (Cat. × Sex)	*p* (Cat. × Role)	*p* (Cat. × Sex × Role)
Stress P.	5.22 ± 3.09	4.55 ± 2.96 *	4.00 ± 2.84 *†	<0.001	0.022	0.889	0.955	0.837
Fatigue P.	5.07 ± 3.03	4.45 ± 2.95 *	3.71 ± 2.88 *†	<0.001	0.030	0.295	0.572	0.645
4SQ Score	8.64 ± 8.22	7.24 ± 7.62 *	5.87 ± 6.74 *†	<0.001	0.020	0.712	0.680	0.918
Alcoholic units	1.63 ± 2.00	1.68 ± 2.03	1.57 ± 1.80	0.403	0.001	0.004	0.109	0.586
Perceived health	6.30 ± 1.75	6.95 ± 1.40 *	7.49 ± 1.28 *†	<0.001	0.068	0.010	0.210	0.672
Sleep quality	5.77 ± 2.22	6.28 ± 2.00 *	6.73 ± 1.80 *†	<0.001	0.021	0.726	0.278	0.023
Work performance	6.33 ± 2.30	6.51 ± 2.21	6.74 ± 2.17 *	0.002	0.004	0.489	0.278	0.863

**Abbreviations:** Cat. = category; ηp^2^ = partial eta squared; role = job role; OLS = overall lifestyle score; SD = standard deviation; n = number of participants in each subgroup; P. = perception; 4SQ score = somatic stress-related symptom score; work performance = self-rated work performance. Values are presented as the mean ± SD. The reported means and standard deviations are descriptive, whereas the *p*-values are derived from adjusted ANCOVA models. ANCOVA models included age as a covariate and OLS category, sex, and job role as fixed factors. Reported *p* values refer to the main effect of OLS category and the indicated interactions; ηp^2^ refers to the main effect of the OLS category. Pairwise comparisons between OLS categories were based on model-adjusted estimated marginal means with Bonferroni correction. * *p* < 0.05 vs. poor; † *p* < 0.05 vs. intermediate. Confidence intervals are presented in Table A3.

**Table 3:** Associations of AHA Healthy Diet score and physical activity categories with health variables. Panel (**a**) AHA Healthy Diet score categories. Panel (**b**) physical activity categories.

(**a**)								
	**Poor** **(Score 0–1)**	**Intermediate** **(Score 2–3)**	**Ideal** **(Score 4–5)**	***p*** **(Cat.)**	**ηp^2^**	***p*** **(Cat. × Sex)**	***p*** **(Cat. × Role)**	***p*** **(Cat. × Sex × Role)**
**AHA Healthy Diet Score**	**Mean ± SD** **n = 786**	**Mean ± SD** **n = 2233**	**Mean ± SD** **n = 361**					
Stress P.	4.96 ± 2.99	4.44 ± 2.95 *	3.69 ± 2.90 *†	<0.001	0.011	0.684	0.161	0.220
Fatigue P.	4.82 ± 2.94	4.27 ± 2.97 *	3.31 ± 2.90 *†	<0.001	0.017	0.476	0.043	0.307
4SQ score	8.09 ± 8.12	6.81 ± 7.32 *	5.80 ± 6.85 *†	<0.001	0.008	0.151	0.302	0.657
Alcoholic units	1.75 ± 2.04	1.64 ± 1.95	1.28 ± 1.62 *	0.035	0.002	0.354	0.889	0.465
Perceived health	6.71 ± 1.63	7.07 ± 1.44 *	7.43 ± 1.42 *†	<0.001	0.016	0.423	0.672	0.373
Sleep quality	6.20 ± 2.01	6.35 ± 2.00	6.70 ± 1.97 *†	0.003	0.003	0.553	0.773	0.044
Work performance	6.34 ± 2.13	6.63 ± 2.23 *	6.65 ± 2.32 *	0.021	0.002	0.083	0.726	0.076
(**b**)								
	**Poor** **(0 METs)**	**Intermediate** **(1–599 METs)**	**Ideal** **(≥600 METs)**	***p*** **(Cat.)**	**ηp^2^**	***p*** **(Cat. × Sex)**	***p*** **(Cat. × Role)**	***p*** **(Cat. × Sex × Role)**
**Physical Activity**	**Mean ± SD** **n = 970**	**Mean ± SD** **n = 1007**	**Mean ± SD** **n = 1403**					
Stress P.	5.07 ± 3.08	4.50 ± 2.92 *	4.05 ± 2.86 *†	<0.001	0.018	0.998	0.419	0.744
Fatigue P.	5.01 ± 3.10	4.38 ± 2.90 *	3.74 ± 2.85 *†	<0.001	0.025	0.648	0.409	0.987
4SQ score	8.20 ± 8.05	7.20 ± 7.47 *	6.02 ± 6.96 *†	<0.001	0.010	0.609	0.196	0.865
Alcoholic units	1.43 ± 1.87	1.62 ± 1.94	1.77 ± 1.98 *	0.024	0.002	0.359	0.789	0.751
Perceived health	6.51 ± 1.65	6.97 ± 1.43 *	7.43 ± 1.31 *†	<0.001	0.053	0.025	0.545	0.576
Sleep quality	6.02 ± 2.12	6.37 ± 1.97 *	6.57 ± 1.91 *	<0.001	0.008	0.241	0.805	0.160
Work performance	6.17 ± 2.27	6.54 ± 2.25 *	6.85 ± 2.12 *	<0.001	0.013	0.303	0.193	0.004

**Abbreviations:** Cat. = category; ηp^2^ = partial eta squared; role = job role; AHA = American Heart Association; SD = standard deviation; n = number of participants in each subgroup; P. = perception; 4SQ score = somatic stress-related symptom score; METs = MET·min/week; work performance = self-rated work performance. Values are presented as the mean ± SD. ANCOVA models included age as a covariate and AHA Healthy Diet score or physical activity category, sex, and job role as fixed factors. Reported *p* values refer to the main effect of category and the indicated interactions; ηp^2^ refers to the main effect of the category. Pairwise comparisons between categories were based on model-adjusted estimated marginal means with Bonferroni correction. * *p* < 0.05 vs. poor; † *p* < 0.05 vs. intermediate. confidence intervals are presented in Table A3, and FDR corrections are represented in Table A4.

**Table 4:** Associations of sleep, smoking habits, and BMI categories with health variables. Panel (**a**) sleep categories. Panel (**b**) smoking habits categories. Panel (**c**) body mass index (BMI) categories.

(**a**)								
	**Poor** **(<5 h)**	**Intermediate** **(5–<7 h or ≥10)**	**Ideal** **(7–<10 h)**	***p*** **(Cat.)**	**ηp^2^**	***p*** **(Cat. × Sex)**	***p*** **(Cat. × Role)**	***p*** **(Cat. × Sex × Role)**
**Sleep**	**Mean ± SD** **n = 118**	**Mean ± SD** **n = 1264**	**Mean ± SD** **n = 1998**					
Stress P.	5.70 ± 3.09	4.81 ± 3.00	4.20 ± 2.91 *†	<0.001	0.015	0.941	0.082	0.585
Fatigue P.	5.19 ± 3.24	4.61 ± 3.03	4.04 ± 2.90 *†	<0.001	0.014	0.765	0.023	0.970
4SQ score	9.74 ± 9.27	7.40 ± 7.71 *	6.57 ± 7.17 *†	<0.001	0.008	0.274	0.039	0.456
Alcoholic units	1.43 ± 1.79	1.66 ± 2.01	1.62 ± 1.91	0.896	0.000	0.191	0.601	0.956
Perceived health	6.15 ± 2.09	6.79 ± 1.58 *	7.23 ± 1.36 *†	<0.001	0.027	0.218	0.302	0.572
Sleep quality	4.77 ± 2.80	5.59 ± 2.09 *	6.93 ± 1.64 *†	<0.001	0.104	0.052	0.011	0.232
Work performance	6.39 ± 2.32	6.65 ± 2.20	6.50 ± 2.23	0.187	0.001	0.029	0.841	0.333
(**b**)								
	**Poor** **(Yes)**	**Intermediate** **(Former ≤12 Months)**	**Ideal** **(No)**	***p*** **(Cat.)**	**ηp^2^**	***p*** **(Cat. × Sex)**	***p*** **(Cat. × Role)**	***p*** **(Cat. × Sex × Role)**
**Smoking Habits**	**Mean ± SD** **n = 427**	**Mean ± SD** **n = 60**	**Mean ± SD** **n = 2893**					
Stress P.	4.83 ± 2.87	4.70 ± 3.02	4.42 ± 2.98	0.702	0.000	0.081	0.331	0.263
Fatigue P.	4.59 ± 2.87	4.63 ± 3.03	4.24 ± 3.00	0.721	0.000	0.047	0.568	0.956
4SQ score	8.43 ± 7.91	7.93 ± 7.36	6.77 ± 7.41 *	0.010	0.003	0.931	0.322	0.648
Alcoholic units	1.83 ± 2.12	2.78 ± 2.39 *	1.57 ± 1.90†	<0.001	0.006	0.571	0.251	0.002
Perceived health	6.74 ± 1.60	6.95 ± 1.62	7.07 ± 1.48 *	<0.001	0.005	0.064	0.226	0.749
Sleep quality	6.47 ± 1.99	6.43 ± 1.94	6.33 ± 2.01	0.350	0.001	0.280	0.929	0.014
Work performance	6.42 ± 2.22	6.82 ± 2.11	6.58 ± 2.22	0.347	0.001	0.704	0.761	0.888
(**c**)								
	**Poor** **(≥30 kg/m^2^)**	**Intermediate** **(25–29.9 kg/m^2^)**	**Ideal** **(<25 kg/m^2^)**	***p*** **(Cat.)**	**ηp^2^**	***p*** **(Cat. × Sex)**	***p*** **(Cat. × Role)**	***p*** **(Cat. × Sex × Role)**
**BMI**	**Mean ± SD** **n = 215**	**Mean ± SD** **n = 957**	**Mean ± SD** **n = 2208**					
Stress P.	4.95 ± 3.22	4.16 ± 2.96 *	4.57 ± 2.94 *	<0.001	0.008	0.011	0.259	0.261
Fatigue P.	4.93 ± 3.12	4.01 ± 2.90 *	4.35 ± 2.99 *†	<0.001	0.011	0.074	0.168	0.152
4SQ score	9.52 ± 8.85	5.98 ± 6.78 *	7.19 ± 7.57 *	<0.001	0.010	0.256	0.213	0.868
Alcoholic units	1.32 ± 1.79	1.80 ± 2.03	1.58 ± 1.91	0.131	0.001	0.683	0.715	0.295
Perceived health	6.18 ± 1.69	6.89 ± 1.52 *	7.17 ± 1.44 *†	<0.001	0.021	0.976	0.854	0.484
Sleep quality	6.04 ± 2.07	6.36 ± 2.03	6.38 ± 1.98	0.061	0.002	0.887	0.250	0.810
Work performance	6.30 ± 2.34	6.59 ± 2.29	6.58 ± 2.18	0.249	0.001	0.744	0.508	0.935

**Abbreviations:** Cat. = category; ηp^2^ = partial eta squared; role = job role; SD = standard deviation; n = number of participants in each subgroup; P. = perception; 4SQ score = somatic stress-related symptom score; work performance = self-rated work performance; BMI = body mass index; kg = kilograms; m^2^ = square metres. Values are presented as mean ± SD. ANCOVA models included age as a covariate and sleep, smoking habits, or BMI category, sex, and job role as fixed factors. Reported *p* values refer to the main effect of category and the indicated interactions; ηp^2^ refers to the main effect of category. Pairwise comparisons between categories were based on model-adjusted estimated marginal means with Bonferroni correction. * *p* < 0.05 vs. poor; † *p* < 0.05 vs. intermediate. Confidence intervals are presented in Table A3, and FDR corrections are represented in Table A4.

**Table 5:** Trend test.

	Kruskal–Wallis (H)	*p*	Jonckheere–Terpstra (Z)	*p*
**Overall Lifestyle Score (OLS)**				
Stress P.	71.813	<0.001	−8.406	<0.001
Fatigue P.	96.865	<0.001	−9.885	<0.001
4SQ score	56.963	<0.001	−7.512	<0.001
Alcoholic units	0.450	0.798	0.386	0.699
Perceived health	284.206	<0.001	17.056	<0.001
Sleep quality	92.642	<0.001	9.564	<0.001
Work performance P.	16.513	<0.001	4.075	<0.001
**AHA Healthy Diet Score**				
Stress P.	45.891	<0.001	−6.619	<0.001
Fatigue P.	66.792	<0.001	−7.847	<0.001
4SQ score	25.825	<0.001	−5.087	<0.001
Alcoholic units	11.408	0.003	−2.833	0.005
Perceived health	61.829	<0.001	7.836	<0.001
Sleep quality	15.587	<0.001	3.664	<0.001
Work performance	14.772	<0.001	3.666	<0.001
**Physical Activity**				
Stress P.	64.158	<0.001	−7.992	<0.001
Fatigue P.	101.066	<0.001	−10.124	<0.001
4SQ score	52.044	<0.001	−7.234	<0.001
Alcoholic units	26.819	<0.001	5.146	<0.001
Perceived health	219.094	<0.001	14.929	<0.001
Sleep quality	39.729	<0.001	6.173	<0.001
Work performance	53.852	<0.001	7.295	<0.001
**Smoking Habits**				
Stress P.	8.084	0.018	−2.834	0.005
Fatigue P.	6.659	0.036	−2.567	0.010
4SQ score	20.247	<0.001	−4.499	<0.001
Alcoholic units	20.334	<0.001	−3.201	0.001
Perceived health	17.694	<0.001	4.168	<0.001
Sleep quality	2.553	0.279	−1.564	0.118
Work performance	3.326	0.190	1.317	0.188
**Sleep**				
Stress P.	50.739	<0.001	−6.839	<0.001
Fatigue P.	36.688	<0.001	−6.001	<0.001
4SQ score	19.278	<0.001	−3.816	<0.001
Alcoholic units	1.337	0.513	0.648	0.517
Perceived health	90.196	<0.001	9.446	<0.001
Sleep quality	380.921	<0.001	19.635	<0.001
Work performance	3.593	0.166	−1.427	0.153
**BMI**				
Stress P.	18.092	<0.001	2.026	0.043
Fatigue P.	17.633	<0.001	0.924	0.355
4SQ	38.566	<0.001	1.412	0.158
Alcoholic units	13.259	0.001	−1.101	0.271
Perceived health	96.141	<0.001	8.728	<0.001
Sleep quality	7.905	0.019	1.605	0.109
Work performance	2.876	0.237	0.257	0.797

**Abbreviations:** H = Kruskal–Wallis test statistic; *p* = probability value; Z = standardized Jonckheere–Terpstra test statistic; OLS = overall lifestyle score; P. = perception; 4SQ score = somatic stress-related symptom score; AHA = American Heart Association; BMI = body mass index; work performance = self-rated work performance. The Kruskal–Wallis test assessed differences across poor, intermediate, and ideal categories, while the Jonckheere–Terpstra test evaluated ordered trends. Positive and negative Z values indicate trend direction. All *p*-values are based on two-sided asymptotic significance.

## Data Availability

The data will be uploaded if the paper is accepted for publication. This raw dataset will be accessible upon request because it includes sensitive information. Requests can be addressed to daniela.lucini@unimi.it.

## Abbreviations

The following abbreviations are used in this manuscript:

4SQ	Somatic stress-related symptom score
AHA	American Heart Association
ANCOVA	Analysis of covariance
a.u.	Arbitrary units
BMI	Body mass index
Cat.	Category
CI	Confidence interval
cm	Centimetres
FDR	False discovery rate
H	Kruskal–Wallis test statistic
h	Hours
HC3	Heteroskedasticity-consistent covariance estimator, type 3
IPAQ	International Physical Activity Questionnaire
kg	Kilograms
m	Metres
m^2^	Square metres
MET	Metabolic equivalent
min	Minutes
MV	Moderate-to-vigorous physical activity
n	Number of participants
ηp^2^	Partial eta squared
OLS	Overall Lifestyle Score
P.	Perception
*p*	Probability value
pFDR	Benjamini–Hochberg false discovery rate-adjusted *p*-value
Q–Q	Quantile–quantile
SD	Standard deviation
SPSS	Statistical Package for the Social Sciences
WHO	World Health Organization
Z	Standardized Jonckheere–Terpstra test statistic

## Appendix A

**Table A1:** Definition of poor, intermediate or ideal for each factor considered.

	Poor (Point: 0)	Intermediate (Point: 1)	Ideal (Point: 2)
-AHA Healthy Diet Score (n. of components)	0–1	2–3	4–5
-Physical Activity (MET·min/week)	None	1–599	≥600
-Current smoking	Yes	Former ≤12 months	No
-Sleep (hours/night)	<5	5–<7 or ≥10	7–<10
-BMI (kg/m^2^)	≥30	25–29.9	<25

**Abbreviations:** AHA = American Heart Association; min = minutes; MET = metabolic equivalent; BMI = body mass index; kg = kilograms; m = metres; m^2^ = square metres. Lifestyle components are classified as poor, intermediate, or ideal according to the criteria shown in the table.

**Table A2:** Characteristics of the study population by job role.

Variable	Executives (n = 941)	Middle Managers (n = 1158)	Non-Managerial Employees (n = 1281)
Stress P.	4.07 ± 2.81	4.39 ± 2.95 *	4.86 ± 3.07 *†
Fatigue P.	3.78 ± 2.81	4.14 ± 2.94 *	4.81 ± 3.06 *†
4SQ score	5.47 ± 6.40	6.53 ± 7.21 *	8.55 ± 8.18 *†
Alcoholic units	1.99 ± 2.05	1.67 ± 1.97 *	1.32 ± 1.78 *†
Work performance	6.58 ± 2.18	6.79 ± 2.22	6.35 ± 2.22 *†
Perceived health	7.13 ± 1.42	7.09 ± 1.49	6.90 ± 1.56 *†
Sleep quality	6.30 ± 1.92	6.48 ± 1.96	6.26 ± 2.10 †
AHA Healthy Diet Score [a.u.]	2.30 ± 0.97	2.29 ± 1.00	2.19 ± 1.04 *†
Physical Activity (METs MV) [MET·min/week]	836.17 ± 1009.37	753.12 ± 1043.72	695.74 ± 1024.59 *
Age [years]	53.98 ± 9.53	49.86 ± 9.16 *	45.23 ± 11.77 *†
Weight [kg]	75.34 ± 13.05	72.87 ± 14.37 *	68.32 ± 14.98 *†
Height [cm]	175.05 ± 8.25	172.89 ± 8.89 *	169.47 ± 9.10 *†
BMI [kg/m^2^]	24.48 ± 3.28	24.23 ± 3.52	23.64 ± 4.10 *†
Sex [n (%)]			
– Females	247 (26.2)	485 (41.9) *	824 (64.3) *†
– Males	694 (73.8)	673 (58.1) *	457 (35.7) *†

**Abbreviations:** n = number of participants in each subgroup; SD = standard deviation; P. = perception; 4SQ score = somatic stress-related symptom score; AHA = American Heart Association; a.u. = arbitrary units; MET = metabolic equivalent; MV = moderate-to-vigorous physical activity; min = minutes; BMI = body mass index; kg = kilograms; cm = centimetres; m^2^ = square metres; work performance = self-rated work performance. Continuous variables are presented as the mean ± SD, while sex is presented as n (%). Pairwise comparisons between occupational roles were performed with Bonferroni correction. * *p* < 0.05 vs. executives; † *p* < 0.05 vs. middle managers.

**Table A3:** Adjusted mean differences across overall lifestyle score categories and individual lifestyle components.

**Overall Lifestyle Score (OLS)**	**Poor−Intermediate** **Mean Difference (95% CI)**	***p***	**Poor−Ideal** **Mean Difference (95% CI)**	***p***	**Intermediate−Ideal** **Mean Difference (95% CI)**	***p***	**ηp^2^**
Stress P.	0.697 (0.342 to 1.051)	<0.001	1.265 (0.908 to 1.622)	<0.001	0.568 (0.294 to 0.843)	<0.001	0.022
Fatigue P.	0.606 (0.256 to 0.956)	<0.001	1.405 (1.053 to 1.758)	<0.001	0.800 (0.528 to 1.071)	<0.001	0.030
4SQ Score	1.750 (0.848 to 2.653)	<0.001	3.093 (2.184 to 4.002)	<0.001	1.343 (0.644 to 2.042)	<0.001	0.020
Alcoholic units	−0.014 (−0.255 to 0.227)	1.000	0.087 (−0.155 to 0.329)	1.000	0.101 (−0.086 to 0.287)	0.586	0.001
Perceived health	−0.618 (−0.801 to −0.436)	<0.001	−1.166 (−1.349 to −0.982)	<0.001	−0.547 (−0.689 to −0.406)	<0.001	0.068
Sleep quality	−0.477 (−0.727 to −0.226)	<0.001	−0.871 (−1.124 to −0.619)	<0.001	−0.395 (−0.589 to −0.200)	<0.001	0.021
Work performance	−0.216 (−0.498 to 0.066)	0.200	−0.410 (−0.694 to −0.126)	0.002	−0.194 (−0.413 to 0.024)	0.099	0.004
**AHA Healthy Diet Score**	**Poor−Intermediate** **Mean Difference (95% CI)**	***p***	**Poor−Ideal** **Mean Difference (95% CI)**	***p***	**Intermediate−Ideal** **Mean Difference (95% CI)**	***p***	**ηp^2^**
Stress P.	0.550 (0.228 to 0.872)	<0.001	1.200 (0.726 to 1.673)	<0.001	0.650 (0.243 to 1.057)	<0.001	0.011
Fatigue P.	0.559 (0.241 to 0.878)	<0.001	1.477 (1.009 to 1.945)	<0.001	0.917 (0.515 to 1.320)	<0.001	0.017
4SQ Score	1.313 (0.494 to 2.132)	<0.001	2.439 (1.235 to 3.644)	<0.001	1.126 (0.090 to 2.163)	0.028	0.008
Alcoholic units	0.074 (−0.143 to 0.291)	1.000	0.335 (0.016 to 0.654)	.036	0.261 (−0.014 to 0.535)	0.069	0.002
Perceived health	−0.362 (−0.533 to −0.192)	<0.001	−0.769 (−1.019 to −0.518)	<0.001	−0.406 (−0.622 to −0.191)	<0.001	0.016
Sleep quality	−0.141 (−0.370 to 0.089)	0.426	−0.476 (−0.813 to −0.139)	0.002	−0.335 (−0.626 to −0.045)	0.017	0.003
Work performance	−0.261 (−0.515 to −0.007)	0.042	−0.375 (−0.749 to −0.001)	0.049	−0.114 (−0.436 to 0.207)	1.000	0.002
**Physical Activity**	**Poor−Intermediate** **Mean Difference (95% CI)**	***p***	**Poor−Ideal** **Mean Difference (95% CI)**	***p***	**Intermediate−Ideal** **Mean Difference (95% CI)**	***p***	**ηp^2^**
Stress P.	0.576 (0.249 to 0.904)	<0.001	0.985 (0.680 to 1.290)	<0.001	0.409 (0.109 to 0.708)	0.003	0.018
Fatigue P.	0.602 (0.279 to 0.925)	<0.001	1.170 (0.869 to 1.470)	<0.001	0.567 (0.272 to 0.863)	<0.001	0.025
4SQ Score	0.964 (0.131 to 1.798)	0.017	1.907 (1.132 to 2.683)	<0.001	0.943 (0.181 to 1.705)	0.009	0.010
Alcoholic units	−0.143 (−0.365 to 0.078)	0.362	−0.235 (−0.441 to −0.029)	0.019	−0.092 (−0.294 to 0.111)	0.832	0.002
Perceived health	−0.468 (−0.638 to −0.298)	<0.001	−0.903 (−1.061 to −0.745)	<0.001	−0.435 (−0.590 to −0.280)	<0.001	0.053
Sleep quality	−0.319 (−0.552 to −0.085)	0.003	−0.453 (−0.670 to −0.236)	<0.001	−0.135 (−0.348 to 0.078)	0.389	0.008
Work performance	−0.488 (−0.745 to −0.230)	<0.001	−0.665 (−0.904 to −0.426)	<0.001	−0.177 (−0.412 to 0.058)	0.212	0.013
**Sleep**	**Poor−Intermediate** **Mean Difference (95% CI)**	***p***	**Poor−Ideal** **Mean Difference (95% CI)**	***p***	**Intermediate−Ideal** **Mean Difference (95% CI)**	***p***	**ηp^2^**
Stress P.	0.648 (−0.131 to 1.427)	0.139	1.344 (0.575 to 2.112)	<0.001	0.696 (0.436 to 0.955)	<0.001	0.015
Fatigue P.	0.500 (−0.275 to 1.275)	0.368	1.186 (0.421 to 1.950)	<0.001	0.686 (0.427 to 0.944)	<0.001	0.014
4SQ Score	2.368 (0.379 to 4.357)	0.013	3.390 (1.428 to 5.353)	<0.001	1.022 (0.359 to 1.686)	<0.001	0.008
Alcoholic units	−0.090 (−0.619 to 0.438)	1.000	−0.068 (−0.589 to 0.454)	1.000	0.023 (−0.154 to 0.199)	1.000	0.000
Perceived health	−0.701 (−1.113 to −0.289)	<0.001	−1.146 (−1.553 to −0.739)	<0.001	−0.445 (−0.583 to −0.308)	<0.001	0.027
Sleep quality	−0.873 (−1.396 to −0.351)	<0.001	−2.188 (−2.703 to −1.673)	<0.001	−1.315 (−1.489 to −1.140)	<0.001	0.104
Work performance	−0.255 (−0.874 to 0.364)	0.972	−0.107 (−0.718 to 0.503)	1.000	0.148 (−0.059 to 0.354)	0.261	0.001
**Smoking Habits**	**Poor−Intermediate** **Mean Difference (95% CI)**	***p***	**Poor−Ideal** **Mean Difference (95% CI)**	***p***	**Intermediate−Ideal** **Mean Difference (95% CI)**	***p***	**ηp^2^**
Stress P.	0.088 (−0.899 to 1.076)	1.000	0.132 (−0.246 to 0.510)	1.000	0.044 (−0.889 to 0.977)	1.000	0.000
Fatigue P.	−0.178 (−1.158 to 0.803)	1.000	0.078 (−0.297 to 0.454)	1.000	0.256 (−0.670 to 1.183)	1.000	0.000
4SQ Score	0.337 (−2.168 to 2.842)	1.000	1.187 (0.228 to 2.145)	0.009	0.850 (−1.517 to 3.217)	1.000	0.003
Alcoholic units	−0.889 (−1.547 to −0.232)	0.004	0.183 (−0.069 to 0.434)	0.248	1.072 (0.450 to 1.693)	<0.001	0.006
Perceived health	−0.220 (−0.743 to 0.304)	0.944	−0.341 (−0.542 to −0.141)	<0.001	−0.122 (−0.616 to 0.373)	1.000	0.005
Sleep quality	0.241 (−0.460 to 0.942)	1.000	0.156 (−0.112 to 0.425)	0.488	−0.085 (−0.747 to 0.577)	1.000	0.001
Work performance	−0.296 (−1.073 to 0.481)	1.000	−0.170 (−0.468 to 0.127)	0.510	0.126 (−0.609 to 0.860)	1.000	0.001
**BMI**	**Poor−Intermediate** **Mean Difference (95% CI)**	***p***	**Poor−Ideal** **Mean Difference (95% CI)**	***p***	**Intermediate−Ideal** **Mean Difference (95% CI)**	***p***	**ηp^2^**
Stress P.	0.958 (0.304 to 1.612)	0.001	1.249 (0.638 to 1.860)	<0.001	0.291 (−0.030 to 0.612)	0.090	0.008
Fatigue P.	0.990 (0.343 to 1.637)	<0.001	1.403 (0.799 to 2.008)	<0.001	0.413 (0.095 to 0.731)	0.006	0.011
4SQ Score	3.059 (1.404 to 4.715)	<0.001	3.624 (2.078 to 5.170)	<0.001	0.565 (−0.248 to 1.378)	0.288	0.010
Alcoholic units	−0.318 (−0.757 to 0.122)	.251	−0.345 (−0.756 to 0.065)	0.132	−0.028 (−0.244 to 0.188)	1.000	0.001
Perceived health	−0.631 (−0.975 to −0.288)	<0.001	−0.995 (−1.316 to −0.675)	<0.001	−0.364 (−0.533 to −0.196)	<0.001	0.021
Sleep quality	−0.342 (−0.808 to 0.124)	.236	−0.419 (−0.853 to 0.016)	0.064	−0.077 (−0.305 to 0.152)	1.000	0.002
Work performance	−0.324 (−0.840 to 0.192)	.399	−0.335 (−0.817 to 0.147)	0.288	−0.011 (−0.265 to 0.242)	1.000	0.001

**Abbreviations:** OLS = Overall Lifestyle Score; AHA = American Heart Association; BMI = body mass index; P. = perception; 4SQ score = somatic stress-related symptom score; CI = confidence interval; *p* = probability value; ηp^2^ = partial eta squared; work performance = self-rated work performance. Mean differences and 95% confidence intervals were estimated using ANCOVA models adjusted for age, sex, and occupational role. Pairwise comparisons between poor, intermediate, and ideal categories were adjusted using Bonferroni correction.

**Table A4:** Unadjusted and Benjamini–Hochberg FDR-adjusted *p*-values for lifestyle-category main effects in ANCOVA models.

Variables	OLS	AHA Diet	Physical Activity	Sleep	Smoking	BMI
Stress P.	*p* < 0.001 pFDR < 0.001	*p* < 0.001 pFDR < 0.001	*p* < 0.001 pFDR < 0.001	*p* < 0.001 pFDR < 0.001	*p* = 0.702 pFDR= 0.737	*p* < 0.001 pFDR < 0.001
Fatigue P.	*p* < 0.001 pFDR < 0.001	*p* < 0.001 pFDR < 0.001	*p* < 0.001 pFDR < 0.001	*p* < 0.001 pFDR < 0.001	*p* = 0.721 pFDR 0.739	*p* < 0.001 pFDR < 0.001
4SQ Score	*p* < 0.001 pFDR < 0.001	*p* < 0.001 pFDR < 0.001	*p* < 0.001 pFDR < 0.001	*p* < 0.001 pFDR < 0.001	*p* = 0.010 pFDR = 0.014	*p* < 0.001 pFDR < 0.001
Alcoholic units	*p* = 0.403 pFDR = 0.434	*p* = 0.035 pFDR = 0.047	*p* = 0.024 pFDR = 0.032	*p* = 0.896 pFDR = 0.896	*p* < 0.001 pFDR < 0.001	*p* = 0.131 pFDR = 0.162
Perceived health	*p* < 0.001 pFDR < 0.001	*p* < 0.001 pFDR < 0.001	*p* < 0.001 pFDR < 0.001	*p* < 0.001 pFDR < 0.001	*p* < 0.001 pFDR < 0.001	*p* < 0.001 pFDR < 0.001
Sleep quality	*p* < 0.001 pFDR < 0.001	*p* = 0.003 pFDR = 0.005	*p* < 0.001 pFDR < 0.001	*p* < 0.001 pFDR < 0.001	*p* = 0.350 pFDR = 0.387	*p* = 0.061 pFDR = 0.078
Work performance	*pP* = 0.002 pFDR = 0.003	*p* = 0.021 pFDR = 0.030	*p* < 0.001 pFDR < 0.001	*p* = 0.187 pFDR = 0.225	*p* = 0.347 pFDR = 0.387	*p* = 0.249 pFDR = 0.291

**Abbreviations:** OLS = overall lifestyle score; AHA = American Heart Association; BMI = body mass index; P. = perception; 4SQ = somatic stress-related symptom score; FDR = false discovery rate; *p* = unadjusted probability value; pFDR = Benjamini–Hochberg FDR-adjusted *p*-value; work performance = self-rated work performance. The Benjamini–Hochberg procedure was applied to the 42 main-effect *p*-values to account for multiple comparisons.
